# Editorial: Celebrating women’s contribution to protein folding, misfolding, and degradation in honor of Susan Lindquist (1949–2016)

**DOI:** 10.3389/fmolb.2024.1504059

**Published:** 2024-10-14

**Authors:** Anat Ben-Zvi, Serena Carra, Esti Yeger-Lotem

**Affiliations:** ^1^ Department of Life Sciences, Ben-Gurion University of the Negev, Beer Sheva, Israel; ^2^ Department of Biomedical, Metabolic and Neural Sciences, University of Modena and Reggio Emilia, Modena, Italy; ^3^ Department of Clinical Biochemistry and Pharmacology and the National Institute for Biotechnology in the Negev, Ben-Gurion University of the Negev, Beer Sheva, Israel

**Keywords:** Apj1, HSF1, Hsp90, synuclein alpha, JUNQ, IPod, LeishIF3d, proteostasis

This article Research Topic is dedicated to the memory and legacy of Professor Susan Lindquist (1949–2016), a pioneer in the field of Protein Folding. Through her visionary experiments in yeast, plants, flies, and human cells, Susan Lindquist explored protein folding, misfolding and aggregation, prion mechanisms of propagation, the heat shock response, and how these processes fuel evolution. During her remarkable career, she coined new concepts and connected ideas across disciplines, often establishing new approaches and tools to study protein quality control. Her insights have paved the way for a better understanding of devastating human diseases such as neurodegeneration and cancer and for the development of innovative treatments based on either the potentiation or inhibition of specific protein quality control players.

Born in Chicago, Susan Lindquist was a college student at the University of Illinois at Urbana-Champaign, a graduate student at Harvard University, and earned her PhD in 1976 at the University of Chicago, where she became a faculty member and established her first lab. In 1988, she became an investigator for the Howard Hughes Medical Institute. In 2001, she joined the biology department faculty at the Whitehead Institute for Biomedical Research at MIT in Cambridge, MA, and became the first female Director of the Institute from 2001 to 2004. She remained a member of the Whitehead Institute, an associate member of the Broad Institute of MIT and Harvard, an associate member of the David H. Koch Institute for Integrative Cancer Research, and a biology professor at MIT for the rest of her career.

Susan Lindquist brought together basic and applied scientists, physicians, mathematicians, biologists, bioinformaticians, and chemists. During her 15-year career at the Whitehead Institute alone, she was a dedicated mentor to over 100 postdoctoral fellows, graduate students, and undergraduates, many of them women, guiding them to productive careers in research. This Research Topic on Protein Folding, Misfolding, and Degradation is dedicated to the memory of Susan Lindquist and her ground-breaking research.

Zhu and Cohen review the cell non-autonomous regulation of the protein homeostasis (proteostasis) network by neuronal mechanisms. They describe the varied responses of the proteostasis network to different proteotoxic challenges, the roles of neurotransmitters and neuropeptides in orchestrating proteostasis in distal tissues, and highlight potential clinical implications. Labbadia focuses on the cellular level and specifically the crosstalk between the main heat shock transcription factor (HSF1) and mitochondria. In his review, Labbadia describes this crosstalk and how it could influence disease susceptibility and human health.

Rios et al. provide an overview of the complex HSP90 molecular machine, whose role as a hub for proteostasis and capacitor for morphological evolution and the manifestations of genetic variation in model organisms have been put forward by the work of Susan Lindquist and colleagues. Wickenberg et al. instead explore the therapeutic potential of HSP90 inhibitors for the treatment of cancer, focusing on the role of HSP90 in the immune system’s response to cancer via regulating the expression of the immunological receptor Major Histocompatibility Complex 1 (MHC1).

Moving to another essential chaperone machine in eukaryotic cells, Ganser et al. describe the importance of the HSP70 cochaperone Apj1 in the propagation and clearance of prions. Prions are aggregated misfolded proteins that accumulate in the form of amyloids and can be fragmented with the assistance of the Sis1 and HSP70 chaperones, releasing particles or “seeds” that can be transferred to the daughter cells, where they can either be eliminated or further propagate. The authors identify two sequence elements of the Apj1 cochaperone that are required for either prion elimination or propagation, revealing divergence amongst different yeast JDPs.

Zaichick and Caraveo also focus on the process of protein aggregation and report on the protective effect of the Food and Drug Administration (FDA)-approved compound FK506 towards α-synuclein, whose accumulation is associated with neuronal toxicity in Parkinson’s Disease and other types of neurological disorders grouped under the name of Synucleopathies. Inhibition of calcineurin by FK506 increased the levels of the insulin growth factor (IGF-1) and decreased the levels of the pro-inflammatory cytokine IL-2 in the cerebral cortex and serum of α-synuclein transgenic mice, providing protective effects.

Rolli et al. identify a novel molecular machine required to spatially sequester at nucleus-vacuole junctions misfolded proteins and promote their clearance via an ESCRT-mediated process highlighting the requirement of HSP40s and HSP70s in the sorting of these misfolded proteins to specific compartments (namely, JUNQ and IPOD).

Lastly, Bose et al. study protein translation in the *Leishmania* parasite. *Leishmania* has a network of cap-binding proteins that can form multiple complexes suggested to support the parasite’s adaptation to changing environmental conditions. Bose et al. describe a new non-canonical cap-binding activity for *Leishmania* protein LeishIF3d and its role in driving translation.

